# In-hospital cardiovascular events after liver transplantation: predictors and long-term outcome

**DOI:** 10.1007/s12471-018-1144-y

**Published:** 2018-08-08

**Authors:** N. T. B. Scholte, M. J. Lenzen, B. van der Hoven, W. J. R. Rietdijk, H. J. Metselaar, C. A. den Uil

**Affiliations:** 1 Department of Cardiology, Erasmus MC-University Medical Center, Rotterdam, The Netherlands; 2 Department of Intensive Care Medicine, Erasmus MC-University Medical Center, Rotterdam, The Netherlands; 3 Department of Gastroenterology and Hepatology, Erasmus MC-University Medical Center, Rotterdam, The Netherlands

**Keywords:** Liver transplantation, Cardiovascular events, Postoperative complications, Risk factors

## Abstract

**Introduction:**

Liver transplantation has emerged as a successful therapy for end-stage liver disease. However, cardiovascular mortality is the leading cause of fatality in the postoperative period. The aim of this study was to reveal the prevalence and identify risk factors of early cardiovascular events (CVEs).

**Methods:**

We performed a retrospective study of all consecutive patients who underwent a primary liver transplantation from 1986 to 2017 (*n* = 916). We investigated the occurrence of in-hospital CVEs, their predictors, and short- and long-term outcome.

**Results:**

The prevalence of CVEs was 11%. The adjusted analysis showed that higher age (OR 1.06, 95% CI 1.03–1.09), higher MELD score (OR 1.04, 95% CI 1.01–1.07 CI) and sinus tachycardia at time of screening (OR 3.12, 95% CI 1.45–6.72) were positive predictors for a CVE. Preoperative propranolol use showed a trend towards a higher risk of CVE (OR 1.66, 95% CI 1.00–2.77, *p* = 0.051). In a sub-analysis of patients where echocardiography data were available (*n* = 597), a larger left atrial diameter and a higher E/E′ ratio were related to early CVEs. Ten-year survival in 30-day survivors was favourable (68.6%; 56.0% vs. 69.8% in the CVE+ vs. the CVE-group, respectively, *p* = 0.056).

**Discussion:**

In conclusion, besides known risk factors (age and MELD score), sinus tachycardia (related to the presence of acute liver failure and cirrhosis) was an independent predictor for CVE after liver transplantation.

## What’s new

- This is an observational study on predictors of in-hospital cardiovascular events (CVEs) after liver transplantation (LT).
- The risk of CVEs after LT was associated with preoperative sinus tachycardia (related to the presence of acute liver failure and cirrhosis, no interaction with the use of propranolol), age and MELD score.
- CVEs were more likely in patients with a larger left atrial diameter and higher E/E′, as indirect indicators for left atrial pressure and diastolic dysfunction.
- Preoperative propranolol use at least did not protect against the risk of CVE.
- The occurrence of CVEs in 30-day survivors did not significantly impair long-term outcome.

## Introduction

Liver transplantation is the treatment for end-stage liver disease. Patients who undergo liver transplantation are at risk for early postoperative cardiovascular events (CVE), primarily through physical stress due to baseline altered haemodynamics as well as perioperative cytokine release and further haemodynamic instability [1–3]. Whereas cardiovascular mortality is the leading cause of short-term death, data on early postoperative cardiovascular events and how these impact long-term outcome are scarce [4]. In previous studies, the prevalence of CVE was estimated to be 12% at 30 days [5, 6]. Occurrence of an early CVE may impact long-term survival [6, 7]. Identification of predictors for early CVEs may impact patient selection and perioperative management. The aims of this study were to investigate the occurrence of early CVEs, to find predictors identified at the time of screening and to describe the correlation with long-term survival.

## Methods

We performed a retrospective study in 923 consecutive patients subjected to their first liver transplant at the Erasmus MC, Rotterdam, the Netherlands between October 1986 and January 2017. All candidates for transplantation received a cardiac evaluation, which at least included a complete history, physical examination and electrocardiogram (ECG). In most cases, except in those who had acute liver failure and underwent highly urgent transplantation, also echocardiography was performed. If indicated by the screening cardiologist, additional tests were carried out.

All data were extracted from the existing liver transplantation database, and (if needed) completed with data from the patient record. The database consisted of the following variables: age, gender, history of hypertension, current smoking, diabetes mellitus, dyslipidaemia, cardiovascular history (arrhythmias, valve disease, cardiomyopathy including heart failure, ischaemic heart disease and cerebrovascular accident or transient ischaemic accident), renal impairment (glomerular filtration rate <60, calculated with MDRD), Model of End-stage Liver Disease (MELD) score, and preoperative propranolol use. ECG data were used to assess heart rate and arrhythmias, including sinus tachycardia, and left ventricular hypertrophy. All baseline variables were identified at the time of screening for liver transplantation, except the MELD score, renal impairment and body mass index (BMI), which were calculated at the time of transplantation. Echocardiography data were collected in order to reveal left ventricular dimensions, systolic function, diastolic function, valve disease and pericardial effusion. ECG and echocardiography data were authorised by staff cardiologists without involvement in this research. In case of uncertainties the primary investigator (CU) reviewed the echocardiographic images blinded for outcomes.

The primary endpoint was the occurrence of in-hospital CVEs. CVEs were defined as myocardial infarction, de novo heart failure, arrhythmias (de novo atrial fibrillation or postoperative recurrent supraventricular tachycardia, other ventricular and supraventricular arrhythmias, or bradycardia), cardiopulmonary resuscitation (including pulseless ventricular tachycardia/fibrillation), stroke, or pulmonary embolism, all occurring after transplantation but within the early postoperative period. The early postoperative period was defined as the in-hospital period following transplantation where, for patients who had a hospitalisation duration longer than 30 days, only CVEs occurring within 30 days were included. Results were stratified according to the occurrence of CVE. The secondary endpoint was survival in the short (30 days) and long term. In January 2017, patient survival status was extracted from the Municipal Civil Registries.

Continuous variables are expressed as means with standard deviation (±SD) or median with interquartile range (IQR). Categorical variables are presented as numbers with percentages. Continuous variables are analysed with the unpaired Student’s *t*‑test if normally distributed or the Mann-Whitney U test otherwise. Normality was assessed using the Shapiro-Wilk test. Categorical variables are compared using the Chi-squire test or Fisher’s exact test when appropriate. All baseline variables are screened for an individual association with CVE by univariate binary logistic regression. In addition to age and gender, the multivariate binary logistic regression analysis included all baseline characteristics that indicated a difference between the two groups (*p*-value ≤0.10). The outcomes are presented as adjusted odds ratios (ORs) with 95% confidence interval (CI). Thirty-day and long-term survival was studied using the Kaplan-Meier method, with the log-rank test to evaluate differences between the CVE+ group and the CVE-group. Patients lost to follow-up were considered at risk until their last contact, at which time point they were censored. A Cox-regression model was used in order to adjust for age in long-term survival. To take into account missing values (variables: sinus tachycardia and MELD score), we also performed a sensitivity analysis using multiple imputation to determine outcome differences between the original and the imputed dataset. Results were assumed statistically significant if *p* < 0.05. All data were analysed with SPSS 20.0 software.

This was an observational study. For this study patients were not subjected to any procedures, neither was any mode of behaviour imposed, otherwise than as part of their regular treatment. Therefore, according to Dutch law and by permission of the local medical ethical committee, (repeated) written informed consent for a patient to be enrolled in this study was not required. Besides, all patients signed consent for the collection and use of data as part of a routine procedure to investigate outcomes of transplantation.

## Results

In this study, data from 916 out of 923 patients who underwent liver transplantation were used: 7 patients were excluded due to incomplete data. Indications for transplantation were cholestatic liver disease (35%), cirrhosis (25%), hepatocellular carcinoma (18%), acute liver failure (14%) and other less common liver diseases (10%). During screening, 597 (65.2%) patients underwent echocardiography. In total 167 (18%) patients underwent additional testing: 18 (2%) had right heart catheterisation, 47 (5%) had an exercise test, 90 (10%) had dobutamine stress echocardiography, 2 (0.2%) had contrast echocardiography, 9 (1%) had a Tc-MIBI scan, 7 (0.8%) underwent Holter ECG monitoring, and 4 (0.4%) underwent coronary angiography. Reasons for these additional tests were angina pectoris or palpitations (11%), exercise tolerance unclear from the history (0.6%), a history of cardiovascular disease or cardiovascular risk factors (30%), ECG or echocardiography abnormalities (13%), other research purposes not related to the current study (35%), or unknown. The additional tests identified 3 patients with mild pulmonary hypertension (mean pulmonary artery pressures 25–30 mm Hg) and 2 patients with coronary ischaemia who were managed medically.

Of 916 patients, 100 (11%) experienced a CVE. Identified CVEs were: myocardial infarction (13%), heart failure (3%), arrhythmias (34%), cardiopulmonary resuscitation (28%), stroke (9%), venous thromboembolism (11%), pericardial tamponade (1%) and hypertensive crisis (1%).

Baseline characteristics are presented in Tab. 1. Patients with a CVE event were older (54 ± 11 vs. 48 ± 13, *p* < 0.001), more frequently had hypertension (24% vs. 15%, *p* = 0.01), had more renal failure (36% vs. 21%, *p* = 0.001), and had a higher BMI (26 ± 5 vs. 25 ± 4, *p* = 0.03). In addition, sinus tachycardia was more frequently present (12% vs. 5%, *p* = 0.005) and the MELD score (20 [15–28] vs. 17 [13–24], *p* = 0.007) was higher. Only 1 patient with preoperative atrial fibrillation and 1 patient with paroxysmal atrial tachycardia had a postoperative event of supraventricular tachycardia. The patient who had a pacemaker did not have a CVE. Sinus tachycardia was often present in patients with acute liver failure (45.3%) and in patients with cirrhosis (30.2%). The 5 patients who underwent additional testing did not have a cardiovascular event after liver transplant.

**Table 1:** Baseline characteristics

	All patients	CVE+	CVE−	Missing	*p*-value
	(*n* = 916)	(*n* = 100)	(*n* = 816)		
Age, years, mean ± SD	48.8 ± 12.5	54.2 ± 10.7	48.2 ± 12.5		**<0.001**
Male sex, *n* (%)	552 (60.3)	65 (65.0)	487 (59.7)		0.31
*Cardiovascular risk factors*					
History of hypertension, *n* (%)	143 (15.6)	24 (24.0)	119 (14.6)		** 0.014**
Current smoking, *n* (%)	172 (23.2)	16 (20.0)	156 (23.6)	176	0.47
Dyslipidaemia, *n* (%)	53 (6.3)	6 (5.9)	47 (6.3)	70	0.87
Diabetes mellitus, *n* (%)	143 (15.6)	22 (22.0)	121 (14.8)		0.062
*History of cardiovascular disease, n (%)*	80 (8.7)	13 (13.0)	67 (8.2)		0.11
Ischaemic heart disease	15 (1.6)	4 (4.0)	11 (1.3)		0.071
Cardiomyopathy	4 (0.4)	0 (0)	4 (0.5)		1.00
Valve disease	10 (1.2)	0 (0)	10 (1.1)		0.61
Arrhythmias	35 (3.8)	6 (6.0)	29 (3.6)		0.23
CVA or TIA	5 (0.5)	1 (1.0)	4 (0.5)		0.44
Other^a^	5 (0.5)	2 (2.0)	3 (0.4)		0.10
Renal impairment, *n* (%)^b^	205 (22.4)	36 (36.0)	169 (20.8)	2	** 0.001**
BMI (kg/m^2^; mean ± SD)	25.3 ± 4.3	26.2 ± 4.5	25.2 ± 4.3	6	** 0.033**
Angina pectoris *n* (%)	14 (1.6)	1 (1.0)	13 (1.6)	17	1.00
*Electrocardiography, n (%)*					
Sinus tachycardia	53 (5.8)	12 (12.0)	41 (5.0)	77	** 0.005**
Left ventricular hypertrophy	73 (9.4)	7 (8.2)	66 (9.5)	136	0.71
Arrhythmias or AV-block	29 (3.2)	6 (6.0)	23 (2.8)		0.086
Atrial fibrillation	14 (1.5)	4 (4.0)	10 (1.2)		0.057
First degree AV-block	11 (1.2)	1 (1.0)	10 (1.2)		1.00
Other^c^	4 (0.4)	1 (1.0)	3 (0.4)		0.37
Pathological Q‑wave, *n* (%)	1 (0.1)	1 (1.0)	0 (0)		0.11
MELD score at time of transplant, median (IQR)	18 (13–25)	20 (15–28)	17 (13–24)	40	** 0.007**
Propranolol use, *n* (%)	205 (22.4)	29 (29.0)	176 (21.6)		0.092
*Aetiology of liver disease, n (%)*					
Acute liver failure	127 (13.9)	18 (18.0)	109 (13.4)		0.21
NASH	16 (1.7)	1 (1.0)	15 (1.8)		1.00

*BMI* body mass index; *CVA* cerebrovascular accident; *CVE* cardiovascular event; *MELD score* Model for end-stage liver disease; *NASH* non-alcoholic steatosis hepatitis; *TIA* transient ischaemic accident

^a^Pericarditis, congenital heart disease and aortic aneurysmatic disease

^b^Renal impairment: MDRD, eGFR <60 ml/min/1.73 m^2^

^c^Paroxysmal atrial tachycardia (*n* = 1), paced rhythm (*n* = 1), atrial rhythm (*n* = 2)

Echocardiography data (available in *n* = 597, 65%) are presented in Tab. 2. Patients with a cardiovascular event had a larger left atrial (LA) diameter (44 ± 5 vs. 41 ± 7, *p* = 0.02) and a greater E/E′ ratio (11 ± 3 vs. 10 ± 3, *p* = 0.02). Patients with cirrhosis had a significantly higher preoperative E/E′ ratio (11 ± 3 vs. 10 ± 3, *p* < 0.001) and LA diameter (43 ± 6 vs. 41 ± 7, *p* < 0.001). There was no relation between LA diameter or E/E′ and the presence of sinus tachycardia.

**Table 2:** Echocardiography data

	All patients	CVE+	CVE−	Missing	*p*-value
	(*n* = 597)	(*n* = 64)	(*n* = 533)		
Estimated systolic PA pressure (mm Hg; mean ± SD)	27.7 ± 8.2	27.3 ± 9.8	27.8 ± 8.0	311	0.76
*Left ventricular function, n (%)*				7	0.10
Good	564 (95.4)	65 (91.5)	499 (96.0)		
Moderate	27 (4.6)	6 (8.5)	21 (4.0)		
Interventricular septum (mm; mean ± SD)	9.5 ± 2.1	9.9 ± 2.8	9.4 ± 2.0	209	0.25
Posterior wall thickness (mm; mean ± SD)	8.9 ± 1.6	9.3 ± 1.6	8.9 ± 1.6	216	0.12
Left ventricular end-diastolic diameter (mm; mean ± SD)	49.8 ± 6.9	49.7 ± 6.3	49.8 ± 6.9	208	0.96
LV mass (g; mean ± SD)	164 ± 54	172 ± 47	163 ± 54	219	0.33
Left atrial diameter (mm; mean ± SD)	41.5 ± 6.4	43.7 ± 5.2	41.2 ± 6.5	198	**0.02**
E/A ratio (mean ± SD)	1.3 ± 0.4	1.2 ± 0.5	1.3 ± 0.4	186	0.93
E/E′ ratio (mean ± SD)	9.9 ± 3.1	11.1 ± 3.1	9.8 ± 3.1	254	**0.02**
*Valve disease, n (%)*				3	
Aortic insufficiency	4 (0.7)	2 (2.8)	2 (0.4)		0.07
Aortic stenosis	2 (0.3)	0 (0)	2 (0.4)		1.00
Mitral insufficiency	9 (1.5)	2 (2.8)	7 (1.3)		0.30
Tricuspid insufficiency	8 (1.3)	1 (1.4)	7 (1.3)		1.00
Pericardial effusion, *n* (%)	13 (2.6)	1 (1.8)	12 (2.7)	103	1.00

**Fig. 1:**
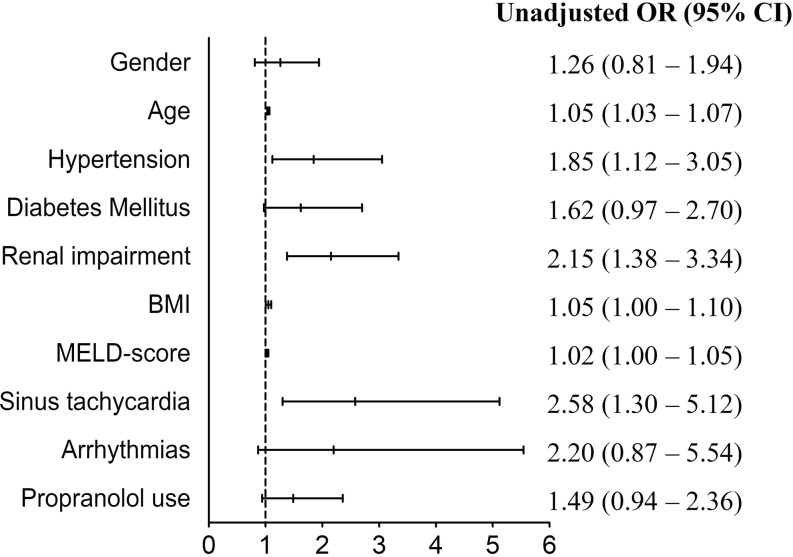
Univariate analysis. *MELD score* Model for End-stage Liver Disease

**Fig. 2:**
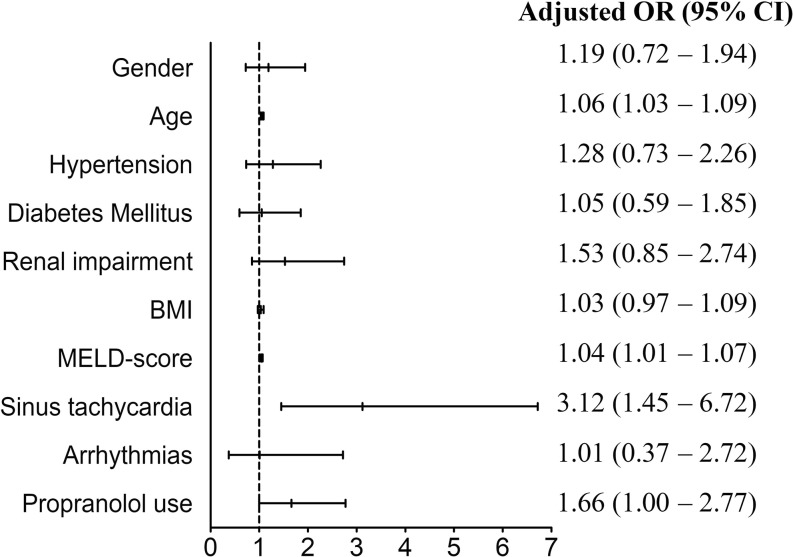
Multivariate analysis. *MELD score* Model for End-stage Liver Disease

The results of univariate analysis are presented in Fig. 1. In multivariate analysis, we found that a higher age (OR 1.06, 95% CI 1.03–1.09), higher MELD score (OR 1.04, 95% CI 1.01–1.07 CI) and sinus tachycardia (OR 3.12, 95% CI 1.45–6.72) were positive predictors for a CVE (Fig. 2). Propranolol use (administered for the treatment of cirrhotic portal hypertension and/or oesophageal varices) showed a trend towards significance (OR 1.66, 95% CI 1.00–2.77, *p* = 0.051). The following interaction terms were calculated and found not significant: propranolol use—sinus tachycardia, propranolol use—MELD score, propranolol use—postoperative atrial fibrillation, and propranolol use—hypertension. After imputing missing values and repeating the analysis mentioned above, no differences were observed. Hence, we present the original data.

**Fig. 3:**
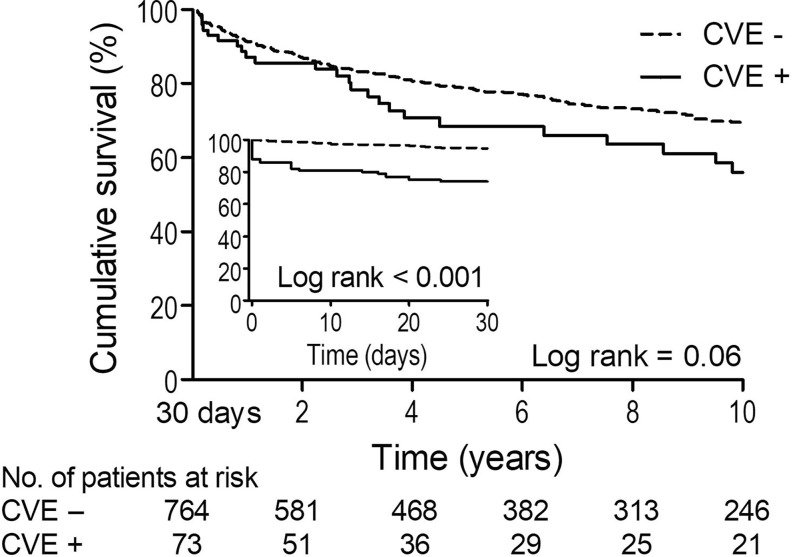
30-day survival and long-term survival. *CVE* Cardiovascular event

Within the first 30 days after liver transplant, there was a lower survival rate (74% vs. 95%, log rank <0.001) in the CVE+ versus the CVE− group, respectively. There was no difference in overall 30-day survival in patients with sinus tachycardia compared with those without sinus tachycardia. Median follow-up time in 30-day survivors was 4.9 years (IQR: 1.3–10.0 years). At 10 years, 209 patients of 30-day survivors (31%) had died. Patients with a cardiovascular event (CVE+) did not have a significantly lower survival rate (log rank = 0.06) at 10 years compared with the CVE− group (Fig. 3). Long-term survival adjusted for age was not different between the two groups (*p* = 0.21).

## Discussion

This study aimed to identify predictors of early CVEs after liver transplantation and the association with short- and long-term outcome. Our main finding was that the risk of a CVE after transplant was associated with sinus tachycardia (without obvious interaction with the use of propranolol), age and MELD score. In a subgroup of patients, LA diameter and E/E′ were related to CVE. The occurrence of CVE did not significantly impact long-term mortality in 30-day survivors.

The presence of sinus tachycardia was an independent predictor for CVE. In the literature, we found no previous evidence for the association between sinus tachycardia and CVE after liver transplantation. A possible explanation might be that patients with sinus tachycardia frequently had acute liver failure (45.3%). Acute liver failure causes vasodilatation, low vascular resistance and hypotension, with a compensatory increase of heart rate [8]. In addition, patients with acute liver failure may have a higher perioperative mortality in comparison with more chronic indications for liver transplant [9], although this was not confirmed by our data. Another explanation might be that the presence of sinus tachycardia preferentially selected patients with cirrhosis (30.2%). Some patients with cirrhosis may have cirrhotic cardiomyopathy, characterised by diastolic dysfunction (as confirmed by our data), which may cause sinus tachycardia in the compensatory phase [10]. A postoperative increase in peripheral vascular resistance, together with changes in preload may precipitate perioperative and postoperative heart failure [11]. Other confounding factors such as thyroid disorders were not assessed in this study.

In line with previous studies, older patients were more likely to experience a CVE [7, 12, 13]. Additionally, we found that a higher MELD score was associated with a CVE, which is also in agreement with a previous study [14].

Preoperative use of propranolol tended to independently predict CVE, in contrast to reports by others [5, 15]. Beta-blocker use in general may be associated with postoperative adverse outcome in patients undergoing major surgery [16]. Second, patients who use propranolol are patients with cirrhosis and, as indicated above, these patients may have cirrhotic cardiomyopathy and diastolic dysfunction [17]. A previous study showed that diastolic dysfunction is associated with early heart failure after liver transplantation [18].

Some variables did not predict CVE. First, renal impairment showed predictive value in univariate analysis but not in multivariate analysis. This finding is in contrast with another study which suggested that patients with renal impairment have an independent risk of CVEs [19]. Also, non-alcoholic steatosis hepatitis was not associated with CVE, which contradicts a previous study [20]. Finally, Dare et al. suggested that BMI could be a risk factor for having CVEs post liver transplant [21]. This finding could not be reproduced either.

In the current study, 10-year survival analysis revealed a slightly higher mortality rate in 30-day survivors in the CVE+ group, although not statistically significant. A larger multicentre study showed a significantly higher mortality rate in the event group [7]; however, we excluded patients who died within the first 30 days.

The major limitation of this study was its retrospective nature, which accounts for the missing data. Missing echocardiographic data are explained by the fact that the transplantation was performed in a highly urgent (ICU) setting, or patients were screened in another university hospital and echocardiographic images/full reports were not sent to our hospital, or echocardiographic data were lost over time. Due to missing data, echocardiographic parameters could not be entered into the multivariate analysis. A further limitation is that the ECG represented a snapshot, where no routine supine resting period was standardised and longer recordings were not routinely performed. Finally, LA diameter was obtained to estimate LA size, where volumes were not routinely measured.

This study presents risk factors and long-term outcome in patients who experience a CVE after liver transplantation. Besides known risk factors (age and MELD score), we identified other less obvious risk factors (sinus tachycardia and maybe the use of propranolol) for the occurrence of CVE. In addition, CVE was more likely in patients with a larger LA diameter and higher E/E′ as indirect indicators for LA pressure and diastolic dysfunction. Long-term survival was not significantly worse in patients who survived an early CVE. Cardiologists should be aware of the higher postoperative risk in a patient with preoperative sinus tachycardia. The obvious suggestion from these data to administer beta-blockers to lower CVE risk is clearly not justified by our data. It might be of value to better investigate markers of diastolic dysfunction at the time of screening to identify higher risk patients. How these findings should change management strategies will be the topic of further research.
